# Editorial: Portal hypertension in cirrhosis and liver vascular diseases: from ethiopathogenesis to current strategies

**DOI:** 10.3389/fmed.2023.1266331

**Published:** 2023-09-11

**Authors:** Xuefeng Luo, Hong You, Fei Gao, Marta Magaz

**Affiliations:** ^1^Division of Gastroenterology and Hepatology, Sichuan University-University of Oxford Huaxi Joint Centre for Gastrointestinal Cancer, West China Hospital, Sichuan University, Chengdu, Sichuan, China; ^2^Beijing Friendship Hospital, Liver Research Center, Capital Medical University, Beijing, China; ^3^Departments of Minimally Invasive and Interventional Radiology, Sun Yat-sen University Cancer Center, Guangzhou, China; ^4^Barcelona Hepatic Hemodynamic Laboratory, Liver Unit, Hospital Clínic Instituto de Investigaciones Biomédicas August Pi i Sunyer (IDIBAPS), University of Barcelona, Barcelona, Spain

**Keywords:** portal hypertension, liver vascular diseases, liver cirrhosis, TIPS, systemic inflammation

This Editorial summarizes the contributions to the Frontiers Research Topic “*Portal Hypertension in Cirrhosis and liver vascular diseases: From Ethiopathogenesis to Current Strategies*” with peer-reviewed articles published in Frontiers in Medicine (Gastroenterology).

Chronic liver diseases represent an important entity in the health system on a global scale. In the setting of cirrhosis, most patients develop portal hypertension (PH) and may potentially develop complications such as hepatic encephalopathy, ascites, renal dysfunction, or spontaneous bacterial peritonitis among others, being a major life-threatening complication of PH, and the formation of varices and variceal bleeding. On the other hand, liver vascular diseases represent a minority complication, and given their low frequency, it is a challenge to gather enough knowledge to make a significant advance in their management. In addition, these vascular disorders can cause non-cirrhotic portal hypertension. Indeed, their prognosis mainly depends on their development since the appearance of gastroesophageal varices or ascites is a milestone in their natural history. This fact is relevant because usually, they affect young patients with an otherwise normal life expectancy that could be markedly reduced if not adequately treated. Hence, it is important to expand the knowledge of their pathophysiology, natural history, diagnostic tools, and potential treatments.

The goal of this Research Topic is to provide a platform for potential authors to highlight recent advances in portal hypertension research, both in cirrhosis and in liver vascular disease. In addition, this Research Topic elaborates on new therapeutic strategies and/or interventional techniques in portal hypertension that have the potential to reduce portal pressure and improve outcomes.

Given the development of portal hypertension that leads to a majority of complications associated with cirrhosis, adequate treatment is very important for such patients. Current medical management of portal hypertension is mainly limited to non-selective beta-blockers and somatostatin analogs. Novel medications that alter hepatic vasculature and the Renin-Angiotensin-Aldosterone System, decrease fibrogenesis, hepatic inflammation, and cell death, and affect gut microbiota are being investigated. In this Research Topic, Sakiani et al. reviewed potential medication treatments currently being studied. Statins, anticoagulants, and Phosphodiesterase inhibitors appear to be the most promising treatment options among others.

Propranolol has been widely used for primary and secondary prophylaxis of variceal bleeding in cirrhotic patients. However, systemic exposure to propranolol in patients with cirrhosis is unclear. Kim et al. examined the pharmacokinetics of propranolol in patients with chronic liver disease compared to that in healthy individuals. The portal perfusion was evaluated by H/L ratio using thallium-201 (201TI) per rectal scintigraphy. The positive correlation with the H/L ratio in patients with chronic liver disease was found based on the area under the concentration-time curve to the last measurable time. This suggested that cirrhotic patients had higher systemic exposure to propranolol than healthy subjects or patients with chronic active hepatitis.

Despite the fact that hepatic venous pressure gradient is accurate to monitor acute or chronic response to beta-blockers in patients with liver cirrhosis, its clinical use is limited by its invasiveness, cost, and availability. Llop et al. evaluated the response to beta-blockers with non-invasive techniques. They found that the combination of spleen TE and damping index for predicting poor acute and chronic response to beta-blockers had an AUC of 0.8 (CI 95 0.5–0.9) and 0.8 (CI 95 0.7–0.9), respectively. Spleen stiff and damping index were able to identify patients who had a poor acute or chronic response to beta-blockers.

Transjugular intrahepatic portosystemic shunt (TIPS) is a well-established interventional procedure for symptomatic portal hypertension. The incidence of TIPS-related technical complications varied between different institutions. The present study reported by Yin et al. evaluated the incidence, management, and outcome of major complications in 948 patients who had undergone TIPS using covered stents. The TIPS procedure showed that major complications occurred in 30 (3.2%) patients among whom eight patients died. This cohort with a large sample size demonstrated that the risk of major complications related to the TIPS procedure is relatively low in an experienced center.

Systemic inflammation has been found to be associated with further decompensation and mortality in patients with advanced cirrhosis. However, the role of serum cytokines in patients with cirrhosis undergoing TIPS remains unknown. Portal and hepatic venous blood samples were obtained during the TIPS procedure. IL-17A and CXCL10 levels were higher in the portal than in the hepatic veins, whereas IL-1RA levels were higher in the hepatic veins than in the portal veins (Liu et al.). Multivariate analysis demonstrated that Child–Pugh scores and IL-8 levels in hepatic veins were independent predictors for mortality during long-term follow-up. These results suggest an association between hepatic inflammation and clinical outcomes in patients with cirrhosis treated with TIPS.

Venous thromboembolism (VTE) could be a fatal complication in hospitalized patients. The risk of VTE in patients with cirrhosis is not determined, and more data are required regarding their bleeding risk during hospitalization. In this Research Topic, Cruz Renó et al. conducted a systemic review to compare the risk for VTE in in-hospital patients with or without cirrhosis. The authors found that in-hospital cirrhotic patients are a heterogeneous group of patients who may present both a higher risk for thrombosis and bleeding than non-cirrhotic patients. In addition, the formation of VTE may prolong the hospital stay in patients with cirrhosis.

Finally, Zhang et al. reported a 33-year-old female patient with non-cirrhotic portal hypertension secondary to cholangiointestinal anastomotic stricture after choledochal cyst surgery. Fortunately, the patient responded well to the dilation of cholangiointestinal anastomotic stricture.

Further studies are required to understand the molecular mechanism of portal hypertension and liver vascular disease. Therefore, more individual management using novel medications and endoscopic or interventional procedures would be possible for such patients.

## Author contributions

XL: Writing—original draft. HY: Methodology, Investigation, Writing—review and editing. FG: Investigation, Writing—review and editing. MM: Investigation, Writing—review and editing.

